# Antibodies to SARS-CoV-2 in follicular fluids and their association with assisted reproduction

**DOI:** 10.3389/fimmu.2023.1120328

**Published:** 2023-03-17

**Authors:** Thilo Samson Chillon, Gregor Weiss, Kamil Demircan, Waldemar B. Minich, Michael Schenk, Lutz Schomburg

**Affiliations:** ^1^ Cardiovascular-Metabolic-Renal (CMR)-Research Center, Institute for Experimental Endocrinology, Charité-Universitätsmedizin Berlin, Berlin, Germany; ^2^ Das Kinderwunsch Institut Schenk GmbH, Dobl, Austria; ^3^ Department of Obstetrics and Gynecology, Medical University of Graz, Graz, Austria

**Keywords:** COVID-19, fertility, reproduction, woman’s health, trace elements, selenium

## Abstract

**Introduction:**

Every second woman suffering from infertility asks for medical help. There is public concern that vaccination-induced antibodies (Ab) are negatively associated with fertility. A recent study has demonstrated an association between SARS-CoV-2 vaccination and a lower pregnancy rate in the subsequent 60 days. Consequently, Ab could affect fertility success in assisted reproduction.

**Methods:**

To address this question, we compared fertilization outcomes of vaccinated (n=35) and nonvaccinated (n=34) women. Paired serum samples and multiple follicular fluids (FF) (up to 10 from the same donor) were collected during the course of assisted reproduction and characterized for oocyte quality, the presence of Ab and trace element concentrations.

**Results:**

The results showed a positive correlation of vaccination-induced neutralizing activity of SARS-CoV-2-Ab in serum and FF. On average, Ab concentrations in serum were higher than in the corresponding FF. However, wide variations in SARS-CoV-2 Ab titers were observed between different FF, correlating to trace element levels, even when retrieved from the same donor.

**Discussion:**

Overall, FF contents are highly variable, but no negative association was observed between Ab in serum or FF and fertilization success and oocyte development, supporting the safety of SARS-CoV-2 vaccination during assisted reproduction.

## 1 Introduction

Infertility affects about one in eight women, half of whom seek medical help (1). Couples are classified as infertile if pregnancy has not occurred after twelve months of unprotected sexual intercourse (2, 3). There is growing public concern about the current COVID-19 pandemic and the impact of infection or vaccine exposure on fertility. In particular, there is concern that antibodies (Ab) to SARS-CoV-2 in response to vaccination negatively affect the blood-follicle barrier (BFB), oocyte quality, and fertility success, although there is hardly any scientific evidence to support this assumption (4). Previous studies have detected Ab in follicular fluid (FF), the significance for fertility is unknown (5, 6).

Consistent with public concerns, a recent study has demonstrated an association between SARS-CoV-2 vaccination and reduced pregnancy rates in the subsequent 60 days (7). While this observational study confirms the concerns, mechanistic evidence is lacking, and the relevant pathways for the adverse effects are unknown. One of the potential mediators for these adverse effects are the induced antiviral Ab and their potential passage through the BFB into the FF. This notion is supported by the results of pilot studies reporting detectable Ab titers in selected FF (5, 6). However, FF composition varies, and size, age, and inflammation affect BFB composition and properties (8, 9) and ultimately influence the success of assisted reproductive technology (ART) (10). To obtain a more comprehensive view of the potential passage of Ab through the BFB, paired serum samples with several FF from the same donor were collected in the course of ART and characterized in terms of oocyte quality and Ab concentrations.

## 2 Materials and methods

### 2.1 Study design

The ART outcome of vaccinated (n=35) and non-vaccinated (n=34) women was compared. In addition, an analytical study was conducted on paired serum and FF samples from 20 adult women (n=10 vaccinated; n=10 unvaccinated) in relation to Ab to SARS-CoV-2. A subset of the donors (n=3 vaccinated; n=2 non-vaccinated) provided 10 individual FF on the same day with the paired serum sample. The recommendations of the Declaration of Helsinki were followed, all participants gave written informed consent before analysis, and the Ethics Committee of Medical University of Graz (approval number: 34-186ex21/22; approval date: 23-Feb-2022) had approved the protocol. All data and samples were collected at Kinderwunsch Institut Schenk GmbH, Dobl, Austria (Cohort 5001_12, KIWI Collection), and the biosamples were stored frozen at the certified Biobank of the Medical University of Graz, Austria, under standardized conditions (11). The quantitative measurements of the Ab were conducted by researchers blinded to the clinical information in an analytical laboratory in Berlin, Germany.

### 2.2 Patient recruitment and therapy

The recruited patients attended the fertility clinic for unexplained primary or secondary infertility, polycystic ovary syndrome (PCOS) or male factor infertility. The patients underwent a controlled ovarian hyperstimulation with gonadotropin-releasing hormone (GnRH) antagonist protocol followed by oocyte retrieval as previously described (12). Intracytoplasmic sperm injection (ICSI) was performed on all mature oocytes (M-II oocytes) 4-5 hours after oocyte retrieval according to standard operating procedures.

### 2.3 Measurement of SARS-CoV2 antibody concentration and neutralizing activity

Ab titers to SARS-CoV-2 were determined *via* a sensitive binding assay using a fusion protein consisting of the S1 domain of SARS-CoV-2 fused in frame to the reporter gene of firefly luciferase. The fusion protein was recombinantly expressed in HEK293 cells, purified, incubated with the biosamples, precipitated by protein A and washed, essentially as described earlier (13, 14). The relative Ab concentrations were determined by calculating the binding index (BI) that denotes the fold of signal strength over the background signal from negative control samples. Neutralizing activity of the samples to the binding interaction of virus spike protein to its receptor ACE2 was measured by a commercial kit (SPIA, Spike Protein Inhibition Assay, product code: DKO205/RUO, ids Holdings PLC) (15). Range of neutralizing activity extended from 0-100%, with a neutralizing activity of 30% and above considered as positive inhibition according to the instructions of the manufacturer (ids Holding PLC, now EUROIMMUN AG, Lübeck, Germany).

### 2.4 Assessment of oocyte quality and embryos

Oocyte quality was assessed by maturity grade. Oocyte maturity was determined by observing the oocytes under an inverted microscope to evaluate the stage of maturity, and classified as follows: Germinal vesicle (GV), metaphase I (MI), and metaphase II (MII). Only MII oocytes were inseminated (16). The embryos were evaluated according to the ESHRE Istanbul Consensus criteria (17). The best consensus embryo was transferred. Embryos that were degenerated or blocked and could not develop to the blastocyst stage, or blastocysts with no inner cell mass or very poor trophectoderm were discarded.

### 2.5 Quantitative analysis of trace elements

The concentrations of the serum trace elements copper (Cu), iron (Fe), selenium (Se), and zinc (Zn) were determined by total reflection X-ray fluorescence (TXRF) analysis using a benchtop TXRF spectrometer (S4 T-STAR, Bruker Nano GmbH, Berlin, Germany), as described (18, 19). Briefly, serum samples were spiked with a gallium standard, applied to polished glass slides, and dried at 37°C. The fluorescence spectrum from X-ray activation measured by the TXRF spectrometer yields information on the particular trace element *via* its characteristic emission wavelength and on the concentrations of the trace elements as area under the emission curve. Inter- and intra assay variations were below 10%, as determined with a commercial trace element standard (Seronorm serum standard, SERO AS, Billingstad, Norway).

### 2.6 Statistics

Study participant characteristics and quantitative data of Ab and trace elements from serum or FF samples are presented as median with interquartile range (IQR) for continuous variables or frequencies for categorical variables. Markers of humoral immune response to vaccination in serum or follicular fluid were compared between vaccinated and non-vaccinated women using the Wilcoxon rank sum test. The Kruskal-Wallis test was used to assess differences between more than two groups. The Spearman rank test was used to evaluate the correlation of immune response parameters. R version 4.1.2 in the RStudio environment was used to perform all statistical analyses. All tests were two-sided, and a p value less than 0.05 was considered to indicate statistical significance.

## 3 Results

### 3.1 Anthropometrics and COVID-19 vaccination parameters of the participants

Vaccinated and non-vaccinated women participating in this observational study, the groups were similar in terms of descriptive parameters, serum hormones, and trace elements (**Table 1**). As expected, the two groups of vaccinated (n=10) and non-vaccinated (n=10) subjects differed in SARS-CoV-2 Ab titers and neutralizing activity of their serum and FF samples (**Table 1**; **Supplementary Figure 1**). In women with multiple oocytes, there was a high intra-individual variation of SARS-CoV-2 Ab titers and neutralizing activity in the different FF samples from the same donor (**Table 2**). When comparing different donors, there were high inter-individual differences (**Table 2**). Similarly, the trace element concentrations varied strongly between different FF samples, even when retrieved from the same donor (**Supplementary Table 1**).

**Table 1:** Anthropometrics and vaccination status .

	Vaccination status	
Variable	Non-Vaccinated, n = 10^1^	Vaccinated, n = 10^1^
Age (year)	36.5 (6.2)	34.5 (7.8)
BMI	25.2 (5.7)	23.1 (11.6)
(Missing^#^)	0	1
Smoking	1/10 (10%)	1/10 (10%)
Number of vaccinations	-	2 (0)
Time since last vaccination (days)	-	35.0 (42.0)
Anti Mueller hormone (ng/mL)	3.6 (3.2)	2.4 (2.7)
Follicle stimulating hormone (mIU/mL)	6.1 (1.4)	7.2 (1.5)
Progesterone (ng/mL)	0.1 (0.1)	0.2 (0.2)
Estradiol (pg/mL)	39.3 (36.3)	39.0 (23.9)
Selenium (µg/L), Serum	73.8 (10.8)	69.3 (11.8)
Zinc (µg/L), Serum	846.0 (118.1)	827.6 (210.5)
Copper (µg/L), Serum	1,312.1 (450.8)	1,137.2 (274.2)
Iron (µg/L), Serum	1,160.4 (263.7)	1,241.7 (129.0)
SARS-CoV-2 Antibody (BI), Serum	1.2 (0.2)	406.8 (96.4)
Neutralization activity (%), Serum	24.0 (6.5)	88.2 (22.4)
SARS-CoV-2 Antibody (BI), Follicular fluid*	1.1 (0.7)	403.1 (270.7)
Neutralization activity (%), Follicular fluid*	0.0 (3.2)	62.3 (39.0)
Selenium (µg/L), Follicular fluid*	26.4 (21.6)	21.5 (22.0)
Zinc (µg/L), Follicular fluid*	253.3 (215.2)	322.8 (219.6)
Copper (µg/L), Follicular fluid*	446.6 (512.5)	597.6 (330.0)
Iron (µg/L), Follicular fluid*	247.1 (268.1)	417.8 (322.4)
Infertility pathology		
Abortus habitualis	0/10 (0%)	1/10 (10%)
Azoospermia	1/10 (10%)	0/10 (0%)
Endometriose	1/10 (10%)	0/10 (0%)
Partner positiv on PGT-M	0/10 (0%)	1/10 (10%)
Pathospermia	4/10 (40%)	2/10 (20%)
PCOS	2/10 (20%)	2/10 (20%)
PCOS & Pathospermia	2/10 (20%)	0/10 (0%)
Tubal factor	0/10 (0%)	4/10 (40%)

^1^Median (IQR) or Frequency (%).

*Results calculated by including all follicular fluid samples (Non-Vaccinated: n = 28; Vaccinated: n = 37).

^#^Missing: Number of participants with missing parameter.

**Table 2:** Vaccination status determined in the follicular fluids of patients with ten individual samples.

	Vaccinated			Non-vaccinated	
Variable	Patient 1, n = 10	Patient 2, n = 10	Patient 3, n = 10	Patient 4, n = 10	Patient 5, n = 10
SARS-CoV-2 Antibody (BI), Follicular fluid					
Median (IQR)	417.9 (204.6)	408.6 (223.7)	430.5 (292.5)	1.0 (0.1)	1.0 (0.2)
Range	20.0, 581.0	112.3, 558.6	72.8, 546.7	0.9, 1.7	0.9, 1.7
Neutralization activity (%), Follicular fluid					
Median (IQR)	74.3 (24.0)	47.6 (25.1)	62.0 (32.3)	0.0 (0.0)	0.0 (1.7)
Range	14.0, 97.1	0.0, 64.5	9.1, 83.1	0.0, 1.7	0.0, 6.6

### 3.2 Comparison of SARS-CoV2 antibodies in serum and follicular fluid samples

**Figure 1:**
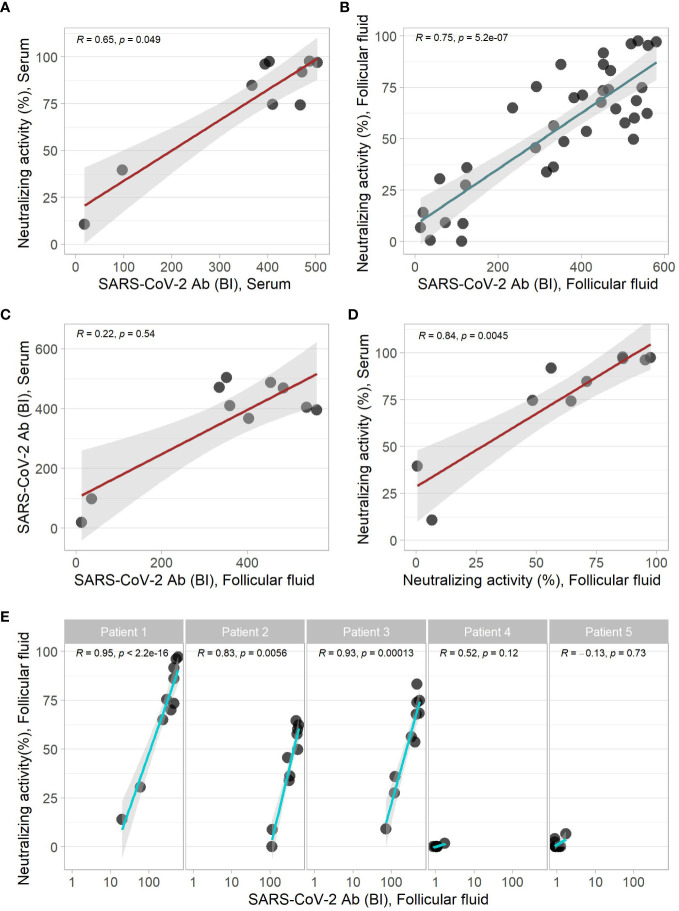
Correlation of COVID-19 parameters of serum and follicular fluid samples. SARS-CoV-2 Ab and neutralizing activity yielded linear correlations in **(A)** serum, and **(B)** the follicular fluid samples. A direct comparison showed **(C)** no significant, or **(D)** a significant correlation of matched serum and folliclular fluid samples in the binding assay and neutralization test, respectively. **(E)** The analysis of different follicular fluids from the same donor indicated significant linear positive correlations of the binding indices and the neutralization results in samples from the vaccinated but not from the non-vaccinated women. Correlation analysis by Spearman`s rank test.

The Ab concentrations to SARS-CoV-2 correlated linearly and strongly to the neutralizing activity in both the serum and FF samples (**Figures 1A, B**). No significant correlation was observed between the concentrations of Ab to SARS-CoV-2 in FF and the paired serum samples (**Figure 1C**). Neutralizing activity of FF and corresponding serum samples correlated linearly (**Figure 1D**). Comparing multiple FF samples retrieved from individual women, linear correlations of neutralizing activity and SARS-CoV-2 Ab concentrations were observed in the individual vaccinated individuals (Patients 1-3, **Figure 1E**), but not in the non-vaccinated subjects with marginal Ab concentrations (Patients 4 + 5, **Figure 1E**).

### 3.3 Variability of COVID-19 vaccination parameters between different follicular fluids

**Figure 2:**
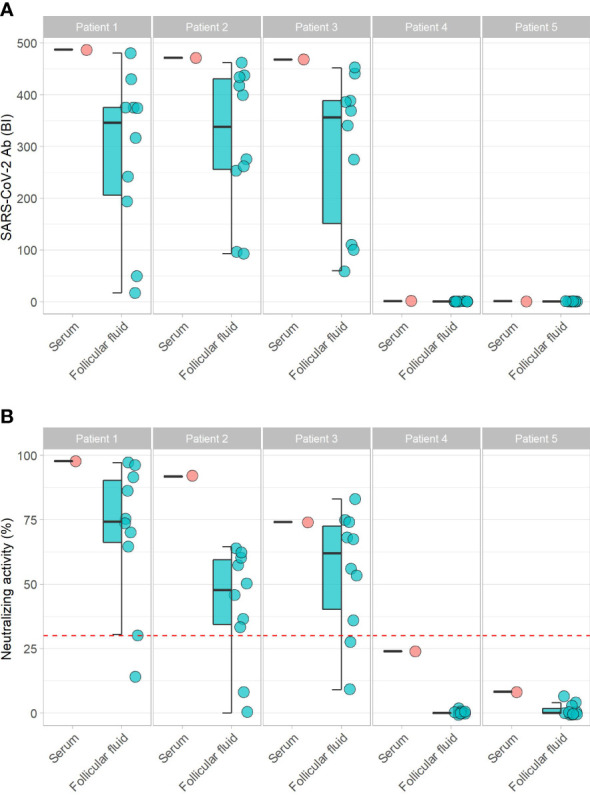
Comparison of SARS-CoV-2 vaccination parameters between paired serum and follicular fluids. SARS-CoV-2 Ab titers differed strongly between different follicular fluids from the same donor, with some samples reaching values as high as in serum **(A)**, whereas others being close to unvaccinated samples, i.e., background. **(B)** Neutralization activities were similar in some follicular fluids to the corresponding serum sample, whereas others were below the threshold for positivity. Collectively, SARS-CoV-2 Ab titers showed high variations in different follicular fluids from the vaccinated women (Patients 1-3), and were low in non-vaccinated samples (Patients 4, 5).

**Figure 3:**
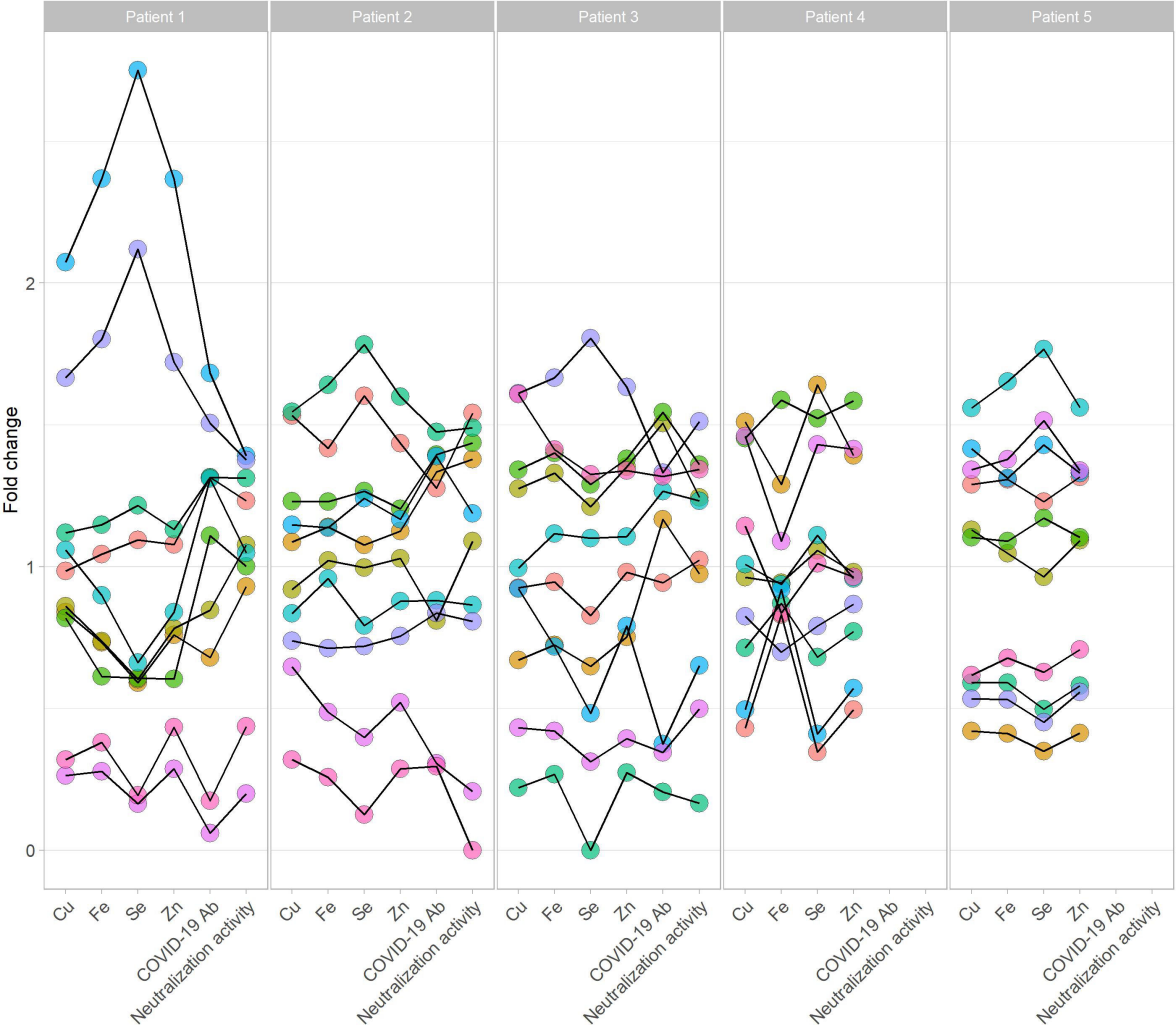
Trace elements and vaccination parameters in follicular fluids from five individual donors. Five donors (left; three vaccinated. right; two non-vaccinated women) provided ten follicular fluids, each labeled by a different color. Relative concentrations in the samples are shown for the trace elements copper (Cu), iron (Fe), selenium (Se), and zinc (Zn), and for the vaccination parameters COVID-19 antibody concentration (COVID-19 Ab) and neutralization activity. The individual dots indicate the relative amount (fold change) to the mean per full data set from all the available samples. In general, trace elements and immunoglobulins were associated in a given follicular fluid, and a follicle with high trace element concentrations also contained on average relatively high vaccination parameters, and vice versa for relatively low values of minerals and antibodies. Colors: Each color stands for one individual follicular fluid of a participant, connected by a thin black line.

SARS-CoV-2 Ab concentration and neutralizing activity showed a high variation in the different FF from the same donor (**Figure 1E**). A direct comparison of the SARS-CoV-2 Ab and the neutralizing activity of individual FF shows a high intra-individual variation of FF to the corresponding serum sample (**Figure 2**). The SARS-CoV-2 Ab concentrations in FF from the same woman varied from high positive reaching almost serum values to almost background and as low as samples from non-vaccinated women (**Figure 2A**). Similarly, neutralizing activity of individual FF from the same women varied from highly positive, similar to the corresponding serum sample, to minimal values, even below the threshold for positivity in the neutralization assay (**Figure 2B**). The quantitative analysis of the trace elements showed strong variations in Cu, Se and Zn concentrations across the samples (**Supplementary Figure 2**). An analysis of all the quantitative data from the different FF retrieved from the same donor indicates that the FF with the relatively highest or lowest trace element concentrations also displayed the highest or lowest SARS-CoV-2 Ab concentrations and neutralizing activities (**Figure 3**).

### 3.4 COVID-19 vaccination parameters in relation to *in vitro* fertilization success

**Figure 4:**
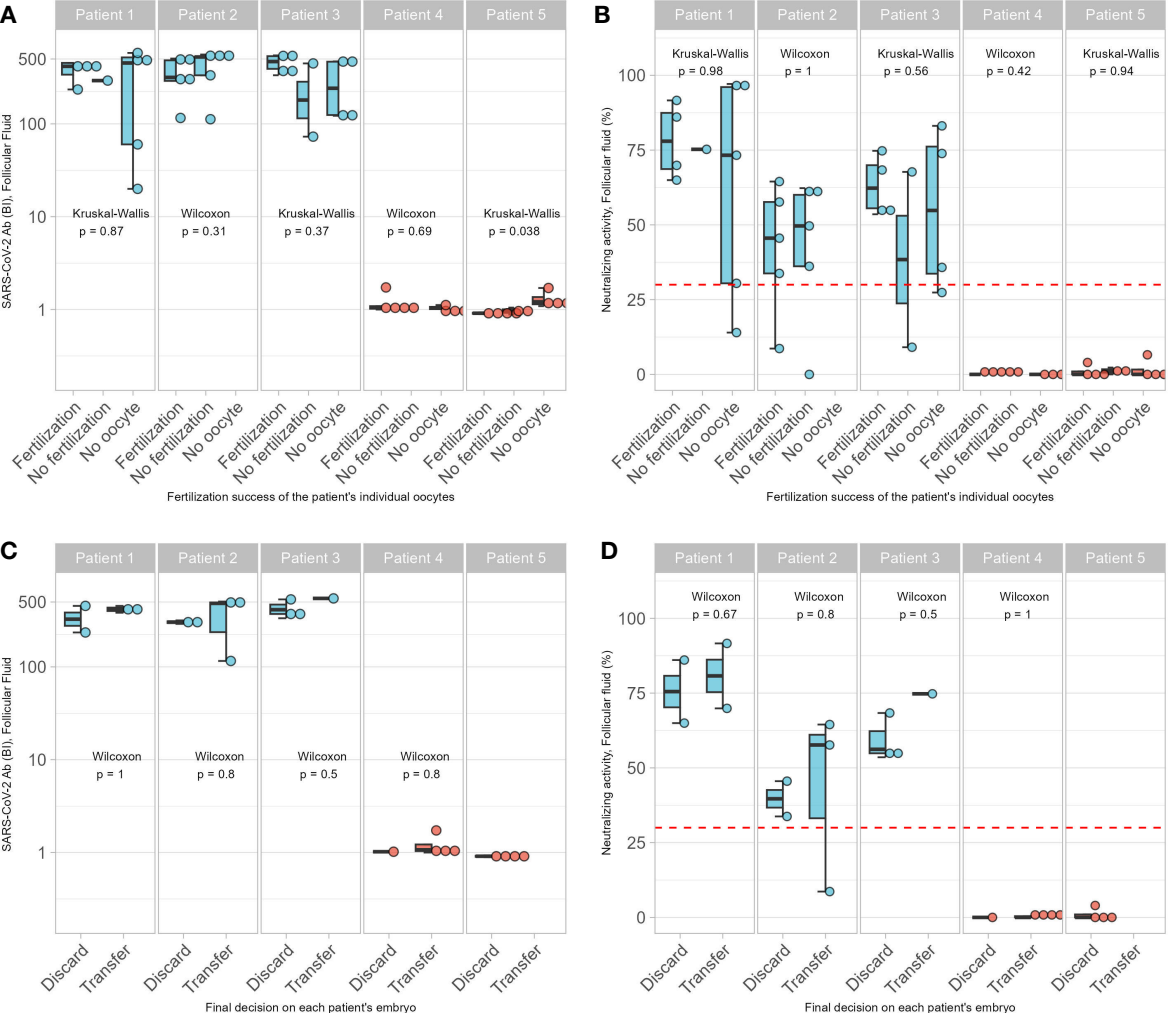
Association of SARS-CoV-2 antibodies on ART. No associations between successful fertilization of oocytes or the number of empty follicles and **(A)** concentrations of SARS-CoV-2 Ab, or **(B)** neutralization activities of the follicular fluids were detected. Similarly, decision to discard or to transfer the oocyte was not associated with **(C)** concentrations of SARS-CoV-2 Ab, or **(D)** the neutralization activities of the follicular fluids from which the oocytes were isolated. Pairwise comparisons were conducted by Wilcoxon rank sum test and Kruskal-Wallis test.

The analysis of the SARS-CoV-2 Ab data with the fertilization success in ART of the retrieved oocytes failed to indicate significant differences between vaccinated and non-vaccinated women (**Figure 4**). Across all the FF, there was no difference in the number of samples without oocyte or in the rate of successful fertilization in relation to the corresponding concentrations of SARS-CoV-2 Ab or the neutralizing activity of the respective FF (**Figures 4A, B**). Similarly, there was no significant difference in the *ad hoc* decision to discard or transfer the embryo in relation to the Ab concentrations or neutralizing activity determined later in the analytical lab (**Figures 4C, D**). Finally, the ART outcome was compared in relation to vaccination. Again, no significant difference was observed for outcome when comparing the vaccinated and non-vaccinated women (**Table 3** **).**

**Table 3:** *In vitro* fertilization outcome of vaccinated and non-vaccinated women.

	Vaccination status		
Variable	Non-vaccinated, n = 44^1^	Vaccinated, n = 45^1^	p-value^2^
**Patient age**	35.41 (5.00)	35.53 (7.00)	0.76
**BMI**	25.93 (9.52)	24.79 (4.05)	0.73
(Missing^#^)	0	1	
**Smoking**	10 (23%)	11 (24%)	0.85
**Outcome**			0.46
Birth	8 (18%)	6 (13%)	
No fertilization	9 (20%)	14 (31%)	
No pregnancy	22 (50%)	21 (47%)	
ongoing	0 (0%)	1 (2.2%)	
Positive hCG	0 (0%)	1 (2.2%)	
Positive hCG, abortion	5 (11%)	2 (4.4%)	

^1^Median (IQR) or Frequency (%).

^2^Wilcoxon rank sum test; Pearson’s Chi-squared test; Fisher’s exact test.

^#^Missing: Number of participants with missing parameter.

## 4 Discussion

In this observational study, we compared the concentrations of vaccination-induced Ab to SARS-CoV-2 between serum and matched FF of women undergoing ART, and tested for adverse effects of the Ab on oocyte quality and fertilization outcome. The two assays used to assess Ab to SARS-CoV-2 yielded largely congruent results, underlying their suitability for the two biological matrices serum and FF. The Ab results were in accordance with the vaccination status reported by the donors. Surprisingly, very strong variations of SARS-CoV-2 Ab titers were observed between different FF, even when retrieved from the same donor at the same time, in line with similarly strong variations in trace element concentrations. The strong association of Ab titers with trace element levels indicates that the variation is not specific for immunoglobulins, but rather a general characteristic of a given oocyte, potentially due to activity level, position within the ovary or maturation stage. On average, the Ab titers were higher in serum as compared to the FF samples. However, the titers in serum and FF were not linearly associated, and an extrapolation from the serum results to the Ab titers in individual FF was not possible. Similarly, the mode of transport for both trace elements and immunoglobulins is not known, and experimental research to characterize the transporters operative at the blood-follicle-barrier is needed. From a functional point of view, no indications for side effects or inhibiting activities of the SARS-CoV-2 Ab on the fertilization success and embryo development were detected, including the *ad hoc* decision to discarding or transferring the embryo. The positive associations of trace element and Ab concentrations in the FF support the notion on the highly variable character of different follicles and indicate some common underlying mechanisms controlling FF composition. Collectively, our results highlight that Ab are capable of passing the BFB, with potential relevance to vaccination, infection, autoimmunity and therapeutic Ab applications, respectively. In case such Ab recognized antigenic structures on the developing oocyte or the surrounding cells within the follicle, adverse effects on oocyte maturation and IVF success may result and warrant a separate analysis.

### 4.1 Intra-individual variability of follicular fluids after COVID-19 vaccination and pregnancy success

A number of observational studies have indicated that there is no higher risk for pregnancy complications after COVID-19 vaccination (20–24). Further, no adverse influence on ART success and embryo quality was observed in relation to COVID-19 vaccination (25, 26), but overall pregnancy rate is reported to be declined during the 60 days after vaccination (7). The database on the presence of Ab to SARS-CoV-2 in FF is small and the available studies focused on individual FF samples per woman and analyzed small numbers of samples only (5, 6). Longitudinal data on IVF success are sparse, and associations of Ab to SARS-CoV-2 with follicular function or ART success were not observed (5). Due to the BFB, there are gradients between plasma constituents and the content of FF (8, 9, 19). This unequal composition is confirmed in the Ab and trace element data presented in this study. However, the differences are not uniform, and a very high variation of the analytes were observed between different FF from the same donor, indicating a highly specific composition of the FF around a given oocyte. Accordingly, an extrapolation from analyses of corresponding serum samples to the FF composition is not possible, and oocyte quality may differ profoundly in case interfering Ab pass the BFB to a variable extent. This notion is of particular relevance for fertility, as the likelihood of developing an autoimmune disease, and hence the likelihood of autoreactive Ab in women of childbearing age is 3- to 10-times higher than for men (27, 28). Circulating autoantibodies (aAb) may account for idiopathic cases of acquired infertility by targeting fertility-relevant factors, like steroids, peptides, proteohormones or their receptors, causing impaired feedback control and loss of regular endocrine signalling in reproductive tissues (29–31). Women with autoimmune diseases such as lupus erythematosus (32), Hashimoto’s thyroiditis (33) or rheumatoid arthritis (34) are known to suffer from reduced fertility (35). The relative contribution to the impaired fertility in autoimmune diseases of a disturbed endocrine axis versus an aAb-mediated adverse effect on the oocyte in a given FF is not conclusively understood. A more comprehensive and systematic analysis of the presence of Ab and aAb in the FF and their potential effect on oocyte quality and embryo development is warranted and may improve the oocyte selection process and the success rate of IVF.

### 4.2 Strengths and limitations

Among the strengths of our analysis is the direct detection of SARS-CoV2 antibodies and neutralizing activity in FF samples from vaccinated and non-vaccinated women in relation to serum, and the comparison of oocyte quality and final ART success. The variability of trace element and Ab concentrations consistently observed across different FF from the same donor constitutes a notable and relevant finding. The limitations of the study include the relatively limited group sizes, and the lack of prospective design. Hence, our results are in need of independent re-evaluations in sufficiently large longitudinal cross-sectional population studies.

### 4.3 Conclusion

The oocyte environment differs considerably between individual follicles of the same woman undergoing ART at the time of oocyte retrieval, both in terms of Ab concentrations and trace element status. While Ab to SARS-CoV-2 were of no direct relevance to IVF and pregnancy success, vaccination appears to be safe with respect to ART. However, Ab that react to the oocyte or follicle cells may affect the success rate of ART, which requires dedicated investigations.

## Data availability statement

The raw data supporting the conclusions of this article will be made available by the authors, without undue reservation.

## Ethics statement

The studies involving human participants were reviewed and approved by Ethics Committee of Medical University of Graz. The patients/participants provided their written informed consent to participate in this study.

## Author contributions

Concept and design: TC, GW, KD, LS, MS. Acquisition, analysis, or interpretation of data: TC, KD, GW, LS. Drafting of the manuscript: TC, KD, GW, LS. Critical revision of the manuscript for important intellectual content: All authors. Statistical analysis: TC, KD. Obtained funding: LS. Administrative, technical, or material support: GW, WM. Supervision: LS, MS, GW. All authors contributed to the article and approved the submitted version.
