# Supplementary material for: Antibodies to SARS-CoV-2 in follicular fluids and their association with assisted reproduction
Source: Front Immunol. 2023 Mar 17;14:1120328. doi: 10.3389/fimmu.2023.1120328 (PMC10064043; doi:10.3389/fimmu.2023.1120328)
Supplement: Supplementary file 1 [file DataSheet_1.pdf]

Supplementary Material SARS-CoV-2 antibodies in assisted reproduction

Supplementary Figures and Tables

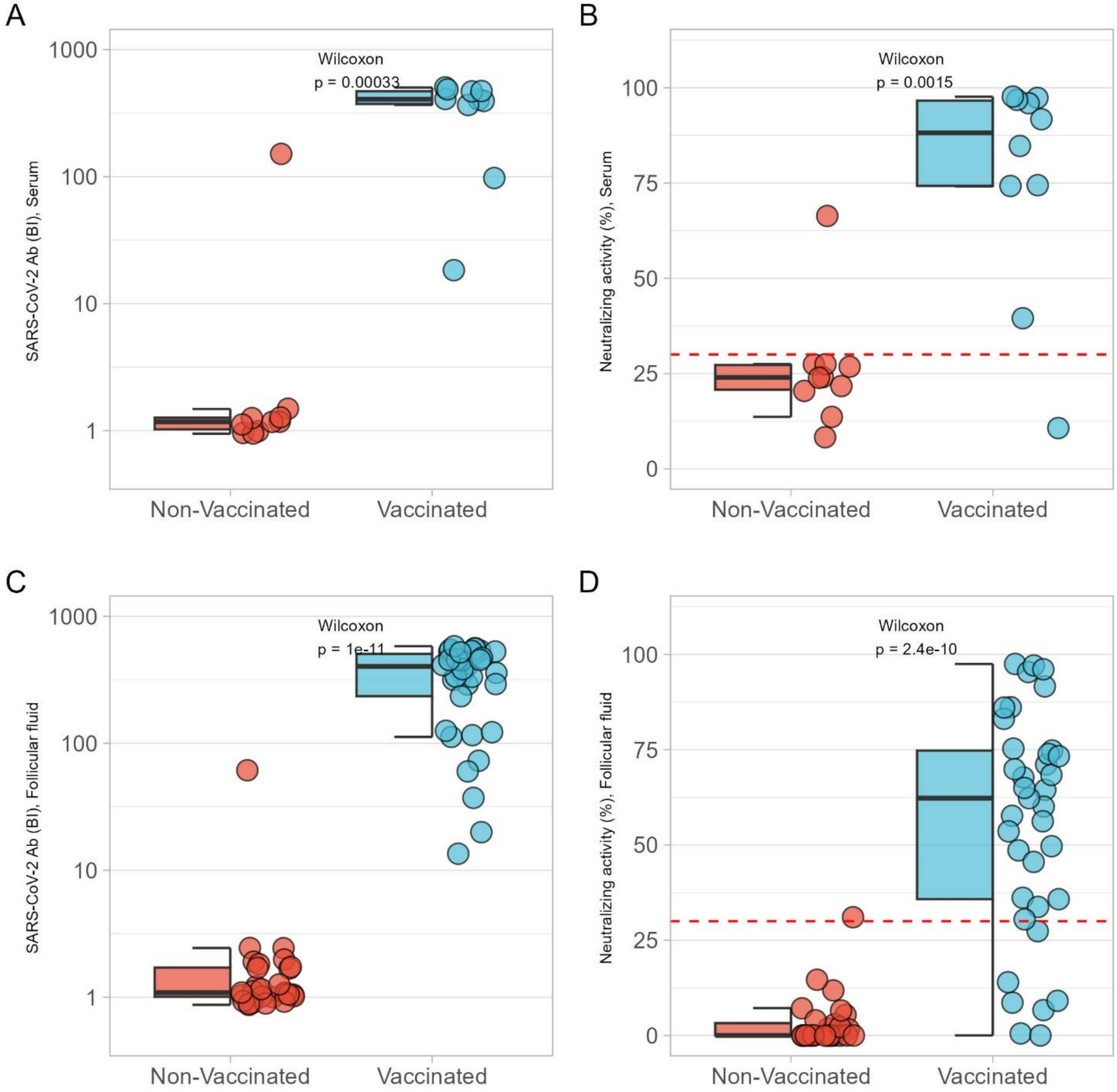

Supplementary Figure 1. Pairwise comparisons of vaccinated and non-vaccinated subjects. A Comparison of serum SARS-CoV-2 antibody titers. **B** Comparison of neutralizing activity of serum samples. **C** Comparison of SARS-CoV-2 antibody titers in follicular fluids. **D** Comparison of neutralizing activity in follicular fluids. Pairwise comparisons were conducted applying Wilcoxon rank sum test. Serum: n= 10; Follicular fluid: Non-Vaccinated: n= 28, Vaccinated: n= 37.

Supplementary Table 1. Trace element status in follicular fluids of patients with ten individual samples

	Vaccinated			Non-vaccinated	
**Variable**	**Patient 1**, n = 10	**Patient 2**, n = 10	**Patient 3**, n = 10	**Patient 4**, n = 10	**Patient 5**, n = 10
**Selenium (µg/L), Follicular fluid**					
Median (IQR)	9.8 (9.2)	26.4 (13.3)	22.6 (17.5)	33.8 (21.0)	23.4 (18.6)
Range	2.6, 42.7	3.2, 45.4	0.0, 42.4	11.3, 53.7	7.7, 38.7
**Zinc (µg/L), Follicular fluid**					
Median (IQR)	158.5 (93.0)	391.0 (148.6)	306.8 (172.6)	204.8 (105.0)	325.8 (212.1)
Range	56.2, 463.2	103.9, 580.4	80.5, 480.4	105.5, 337.6	122.7, 463.0
**Copper (µg/L), Follicular fluid**					
Median (IQR)	351.7 (106.9)	651.6 (290.1)	455.6 (280.7)	325.8 (209.4)	815.6 (534.7)
Range	100.2, 790.4	207.2, 1,004.0	104.3, 764.8	142.3, 499.2	306.6, 1,140.0
**Iron (µg/L), Follicular fluid**					
Median (IQR)	215.7 (126.4)	471.0 (189.4)	412.1 (264.9)	157.6 (35.3)	416.0 (271.9)
Range	73.3, 624.8	112.5, 716.0	106.9, 665.6	118.6, 269.4	160.3, 643.8

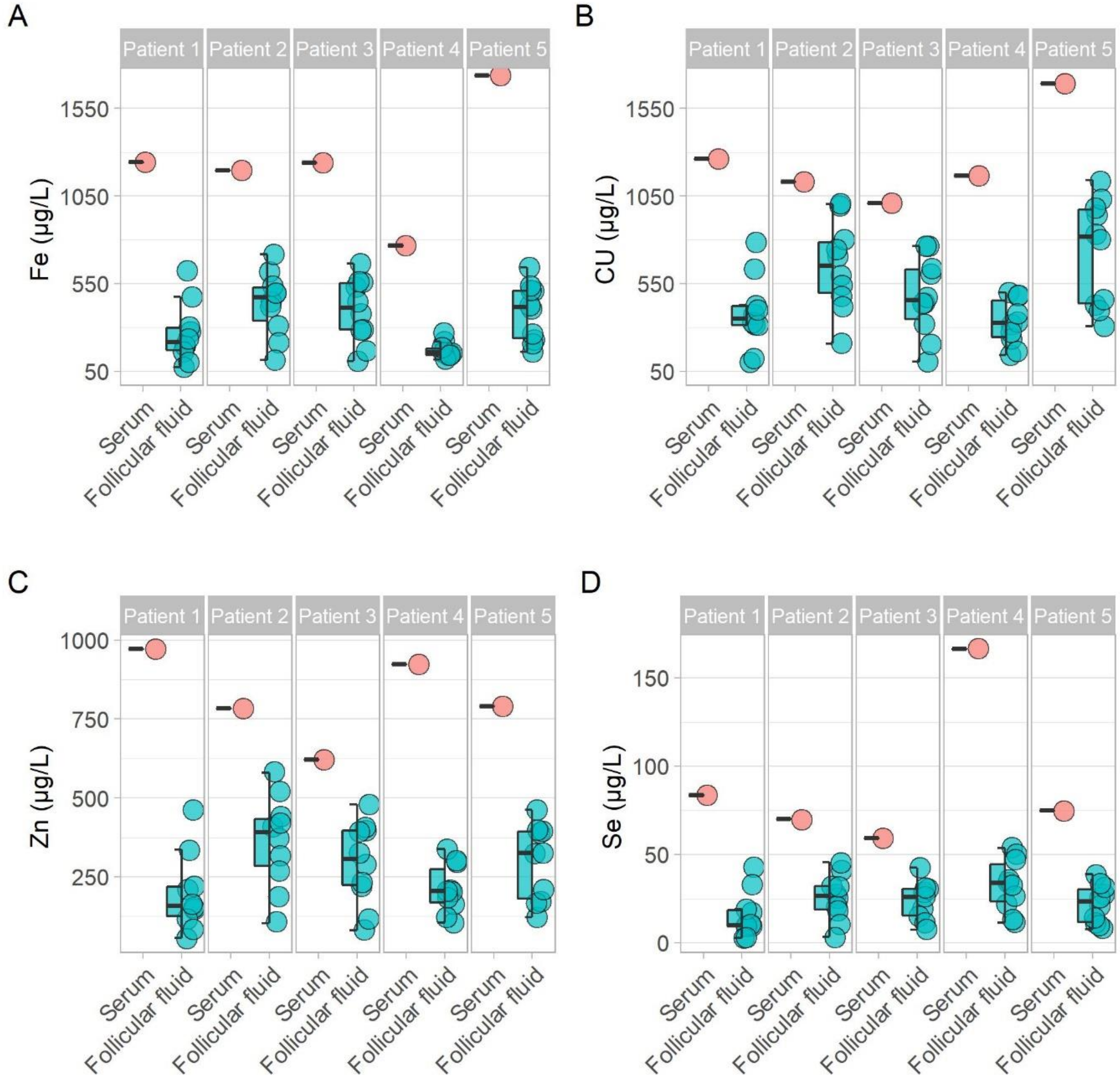

Supplementary Figure 2. Trace element (Iron, Copper, Zinc, Selenium) titers of serum and follicular fluid is shown next to each other for each participant individually.
